# A Fully Automated Synthesis of 14-(*R,S*)-[^18^F]fluoro-6-thia-heptadecanoic Acid ([^18^F]FTHA) on the Elixys Radiosynthesizer

**DOI:** 10.3390/ph17030318

**Published:** 2024-02-29

**Authors:** Usevalad Ustsinau, Lukas Nics, Marcus Hacker, Cecile Philippe

**Affiliations:** Division of Nuclear Medicine, Department of Biomedical Imaging and Image-Guided Therapy, Medical University of Vienna, 1090 Vienna, Austria

**Keywords:** [^18^F]FTHA, fluorine-18, automation, fatty acid metabolism, radiosynthesis

## Abstract

14-(*R,S*)-[^18^F]fluoro-6-thia-heptadecanoic acid ([^18^F]FTHA) is a radiocompound for imaging the fatty acid circulation by positron emission tomography. A revived interest in imaging of lipid metabolism led us to a constant tracer production over three years, initially using a conventional vessel-based synthesizer and later transitioning to the cassette-based Elixys synthesizer. On the Elixys module, the radiochemical yield of [^18^F]FTHA could be increased by more than two times, reaching 13.01 ± 5.63% at the end of the synthesis, while maintaining necessary quality control results.

## 1. Introduction

Metabolic disturbances are implicated in the pathogenesis of numerous diseases. There are several available nuclear medicine tracers for non-invasive assessment of metabolic alterations via positron emission tomography (PET). The most common tracer allowing us to visualize energy metabolism is the fluorinated glucose analogue 2-deoxy-2-^18^F-fluoro-D-glucose ([^18^F]FDG), which is also the gold standard in tumor imaging. However, the glucose metabolism, although important, does not describe all aspects of the nutrient metabolism. For example, free fatty acids (FFAs) are the main source of energy for the myocardium and skeletal muscle [1]. To define that side of the energy metabolism, a radiolabelled fatty acid analogue is required.

The versatility and applicability of covalently bound radionuclides are vast, especially as the chemical structure is not (^11^C) or only slightly (^18^F) altered compared to PET tracers with a metal-based radiolabel [2]. For instance, [1-^11^C]palmitate can be used as a free fatty acid analogue without changing its biochemical properties [3]. However, the short half-life of the isotope and ^11^C-carrying metabolites (especially [^11^C]CO_2_ and [^11^C]HCO_3_^−^) require an increased dosage for metabolic studies of patients and necessitate the assessment of the input function through blood sampling. Hence, significant efforts to improve labelling efficiency were made in the last decade to address the growing importance of nucleophilic ^18^F-fluorination chemistry [4]. With a half-life of 109.7 min, fluorine-18 is more convenient for tracer production and the exploration of metabolic pathways, avoiding the release of ^11^C-metabolites into the circulatory system.

14-(*R,S*)-[^18^F]fluoro-6-thia-heptadecanoic acid ([^18^F]FTHA) is the most used PET radiotracer for assessing fatty acid utilization. The tracer was widely used in studies of the myocardium [5,6,7] in the late 1990s and the beginning of 2000s. Experimental studies in pigs have shown a correlation between trapping of [^18^F]FTHA and fatty acid oxidation in myocardial muscle [7]. However, in hypoxic conditions of the myocardium, [^18^F]FTHA may not be optimal for measuring changes in β-oxidation [8]. Another important role could be in visualizing FFA synthesis and their transfer. The liver and adipose tissue are the main lipogenic tissues. Recent studies show a revival of interest in FFA metabolism, with the application of adipose tissue function [9,10], obesity [11,12,13], and type 2 diabetes [14,15].

The [^18^F]FTHA tracer synthesis was developed in 1991 by DeGrado [16]. Savisto et al. [17] slightly modified the method for automated production of [^18^F]FTHA to make it available for good manufacturing practice (GMP). That method is currently assumed to be the state of the art and is used by other researchers. In our facility at the General Hospital of Vienna, we utilized a conventional vessel-based synthesizer, following the latest instruction for the production of [^18^F]FTHA [17] over a period of two years.

The increased demand for automated radiosyntheses and the advancement in the technological development stimulate a request for new modules [18]. A recent and commercially available module is the Elixys Flex/Chem radiosynthesizer. The main advancement of this module is a cassette-based system, where disposable cassettes carry out different functions such as sealed reactions, evaporations, and reagent addition. A gas handling robot moves sealed reagent vials from storage locations in the cassette to addition positions and dynamically provides a vacuum and inert gas to ports on the cassette [19]. The Elixys has shown its robustness for the automated production of multiple fluorine-18 tracers [19,20,21,22].

After an update of the equipment to the Elixys radiosynthesizer, we have automated the synthesis without the need for substantial modification of the synthesis approach. Our primary goal was to quickly transfer the established synthesis onto the new platform for immediate use in animal trials. Herein, we describe the first demonstration of an [^18^F]FTHA synthesis in the Elixys radiosynthesizer and compare the final yield and purity with syntheses performed in a conventional vessel-based synthesizer.

## 2. Results

In the years 2020–2022, we conducted 46 successful automated syntheses of [^18^F]FTHA on the vessel-based PET-synthesizer (Section 4.2.1), with a radiochemical yield (RCY) of 5.52 ± 2.38% (0.23–4.56 GBq) at the end of the synthesis (EOS), starting from 25–55 GBq of [^18^F]fluoride (Table 1). After the transfer of the [^18^F]FTHA production to the Elixys in 2022–2023 (Section 4.2.2), we performed 12 successful syntheses, with a significantly increased RCY of 13.01 ± 5.63% (1.60–6.27 GBq) at the EOS starting from 19–26 GBq (Table 1).

The radiochemical purity (RCP), as determined by analytical HPLC, exceeded 95% in all syntheses (Table 2). The average pH of both the vessel-based synthesizer and the cassette-based Elixys was 7.1 ± 0.2; the average osmolality was 292 ± 42 mosmol/kg; and the Kryptofix 222 was <5 μg/mL. Gas chromatography revealed < 45 ppm MeCN and <187 ppm MeOH. All syntheses were for either in vitro cell uptake or preclinical in vivo experiments. All quality control parameters were in full accordance with the standards for animal application at the General Hospital of Vienna (Table 2).

## 3. Discussion

The goal of this work was to establish the radiochemical synthesis of [^18^F]FTHA on available automated radiosynthesizers to facilitate access to the imaging agent of FFA metabolism for preclinical research. For that purpose, we utilized a former ^11^C-methylation vessel-based synthesizer and the Elixys Flex/Chem with Pure/Form. The preparation of reagents for the automated synthesis production of [^18^F]FTHA according to Savisto et al. [17] demonstrated its robustness and stable quality control in 58 syntheses over three years of consecutive work. 

We also successfully established a transfer to the new automated module—Elixys Flex/Chem with Pure/Form. To our knowledge, it is the first usage of the Elixys radiosynthesizer for the production of [^18^F]FTHA. Previously, [^18^F]FTHA was reported to be very susceptible to radiolytic oxidation [18]. That factor and/or oxidation by air as the reactor elevates and moves several times in an open space during the synthesis were among the main concerns for the production. Based on our reports and yield results, we can conclude that these concerns have been allayed. Among the other issues we had to manage during the synthesis in the cassette-based Elixys radiosynthesizer was the leakage of fluorinated H_2_^18^O during the trapping of the [^18^F]fluoride on the anion exchange cartridge. Either loose fittings or leakage in the input lines led to the loss of some fluorinated H_2_^18^O, resulting in less activity being trapped in the PS-HCO_3_- and consequently less activity in the reactor.

Analyzing the RCY of [^18^F]FTHA (Table 1), we can conclude that the total amount of formulated end product at the vessel-based synthesizer (2.09 ± 0.99 GBq) was similar to that previously demonstrated by Savisto et al. [17] (1.7 ± 0.8 GBq). Moreover, the results of the RCY at the Elixys module showed an increase to 3.13 ± 1.41 GBq (range of 1.60 to 6.27 GBq at EOS). Comparing the % of RCY between the conventional vessel-based synthesizer (5.52 ± 2.38%, *n* = 46) and the cassette-based Elixys radiosynthesizer (13.01 ± 5.63%, *n* = 12), we discovered a significant increase (according to Student’s *t*-test *p* < 0.001) in yield after a transfer to the Elixys. Notably, this increase was achieved despite a slightly longer duration of the synthesis (~7 min extra). We believe that this is due to the more effective azeotropic drying and the generally highly efficient evaporation stages in the Elixys module. The solvents were evaporated under argon pressure in a sealed reactor, which reduced spillover and other losses. After the first trials, radiosynthesis demonstrated its effectiveness, and we decided to reduce the initial amount of [^18^F]fluoride, resulting in a higher % of RCY and less radiation exposure in the production site.

To this end, the constant successful chemical quality control results after all syntheses (Table 2) confirm the reliability of the synthesis. Our preclinical study [11] has not recorded any difference in the blood uptake and imaging with [^18^F]FTHA produced on either module. Additionally, the automated production in the cassette-based Elixys radiosynthesizer can positively affect clinical studies investigating FFA alterations, as multiple clinical doses could be produced in one synthesis. This could expand and promote the production of the [^18^F]FTHA tracer for future research on nutrients and energy metabolism.

## 4. Materials and Methods

### 4.1. Materials 

The list of chemical reagents, including their product numbers and provider companies, is presented in Table 3. All reagents were used as supplied without further purification for all the syntheses presented in this article. Both precursor and reference standard were stored at −20 °C and are stable for at least 3 years.

The ^18^F separation cartridge PS-HCO_3_- (Synthra, Hamburg, Germany) was used for the ^18^F^−^ trapping. A solid phase extraction (SPE) cartridge (Light C18 Sep-Pak, Waters Corp., Milford, MA, USA) was conditioned with ethanol (10 mL, Table 1) to wet the stationary phase, followed by an equilibration step with sterile water (20 mL, B. Braun), and then used for the final product formulation.

### 4.2. Radiochemistry

**Figure 1:**
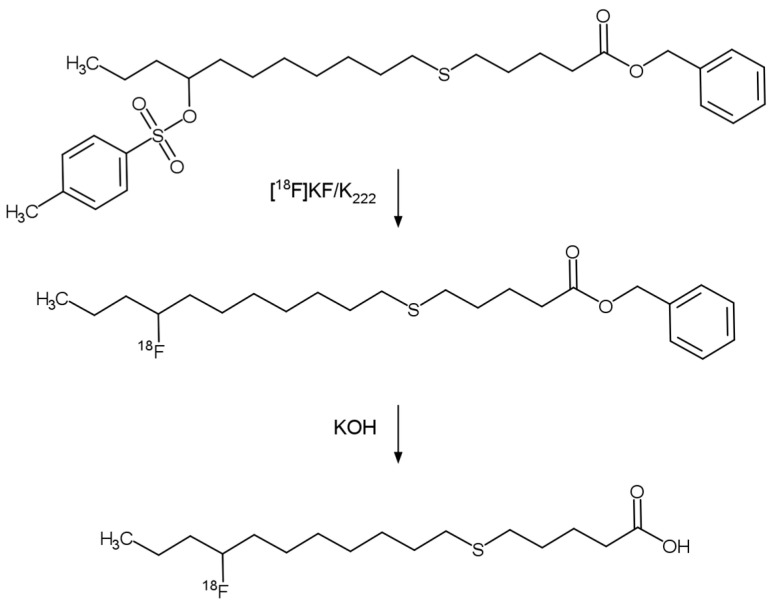
General reaction scheme: precursor (Benzyl-14-(*R,S*)-tosyloxy-6-thiaheptadecanoate) undergoes nucleophilic substitution with fluorine-18 and is further hydrolyzed with KOH to remove the protecting group to yield 14-(*R,S*)-[^18^F]fluoro-6-thia-heptadecanoic acid.

The [^18^F]FTHA tracer production reaction has been fully described by DeGrado [16] and Savisto et al. [17] and includes two general steps: nucleophilic substitution with [^18^F]fluoride in the precursor (Benzyl-14-(*R,S*)-tosyloxy-6-thiaheptadecanoate, Table 3) and hydrolysis with the strong base (KOH, Table 1) to remove the protecting group for yielding 14-(*R,S*)-[^18^F]fluoro-6-thia-heptadecanoic acid, as is shown in Figure 1.

[^18^F]fluoride was produced via the ^18^O(*p,n*)^18^F reaction in a GE PET trace cyclotron (16.5 MeV protons; GE Medical Systems, Uppsala, Sweden). H_2_^18^O (HYOX18; >98%) was purchased from Rotem Europe (Leipzig, Germany). Typical beam currents were 48–52 μA, and irradiation was stopped as soon as the desired activity level was reached (19–55 GBq).

#### 4.2.1. Production of [^18^F]FTHA in the Vessel-Based Synthesizer 

For the automated syntheses, a former ^11^C-methylation vessel-based PET synthesizer (formerly Nuclear Interface, now General Electric Medical Systems, Uppsala, Sweden) was used.

**Figure 2:**
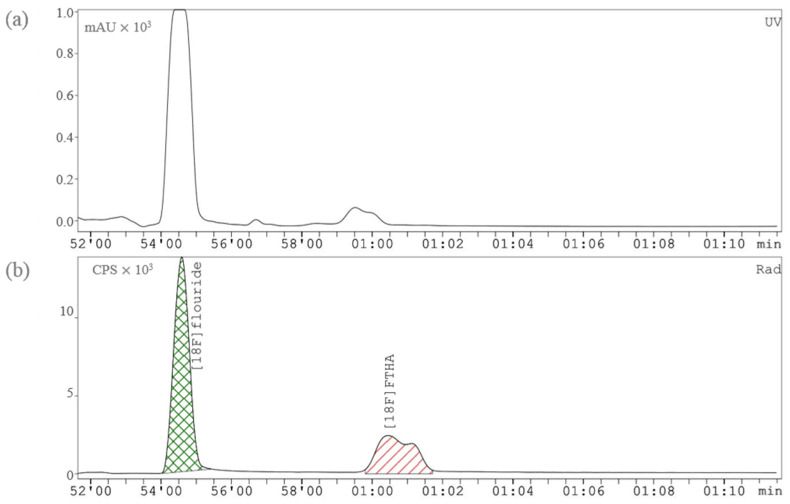
Preparative HPLC chromatograms of [^18^F]FTHA in the vessel-based synthesizer: (**a**) UV channel (230 nm), (**b**) radioactivity channel.

The first step of the synthesis was the trapping of the [^18^F]fluoride (20–55 GBq) on the anion exchange cartridge (PS-HCO_3_-), followed by its release and transfer to the reactor with the elution of Solution A (Table 4). Iterative azeotropic drying was performed at 120 °C by the addition of three times 500 μL dry MeCN. Subsequently, the reactor was cooled to 35 °C, and the dissolved precursor (V1, Table 4) was transferred into the reactor with a constant helium flow of 50 mL/min. The mixture was stirred at 100 °C for 10 min and then at 85 °C for 5 min. 2M KOH (V2, Table 4) was added into the reactor, and the solution was stirred at 90 °C for 5 min. During that hydrolysis reaction, the protection group of the fluorinated intermediate was removed. After cooling to room temperature, Solution B (V3, Table 4) was transferred to the reactor to neutralize the reaction mixture, which was subsequently injected into the built-in HPLC. The preparative HPLC measurements were performed with the HPLC column Gemini 10 μm C18 110Å 250 × 10 mm, Phenomenex (Torrance, CA, USA), using a mobile phase with the ratio of 850:150:4:2 (*v*/*v*/*v*/*v*) MeOH/H_2_O/AcOH/L–Ascorbic acid and on flow rate of 8 mL/min. Average retention time of [^18^F]FTHA was between 6 and 8 min after injection (Figure 2). The product peak was collected into the bulb containing Solution C (Bulb, Table 4), followed by an automated purification and formulation. Therefore, the content of the bulb was passed though the C-18 cartridge into the SPE waste. Then, 10 mL of Solution C (V6, Table 4) was used to wash the C-18 cartridge. The purified product was eluted with 0.8 mL ethanol (V5, Table 4) and further diluted with physiological saline solution (0.9%) into the product collection vial. The last step of the synthesis was the transfer of the product into the sterile final product vial (TechneVial 11 mL, Curium, France), which was prefilled with 4 mL of physiological saline solution containing 8% BSA to achieve a final formulation of 10% EtOH.

#### 4.2.2. Production of [^18^F]FTHA in the Cassette-Based Elixys Synthesizer

**Figure 3:**
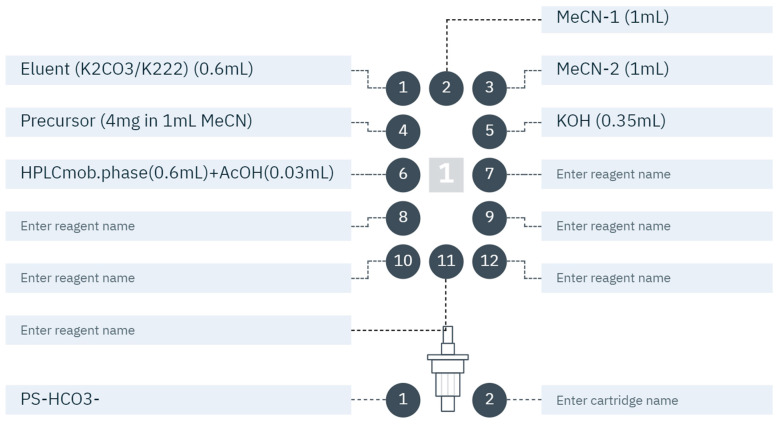
Positions of reagents and ^18^F^−^ separation cartridge in the cassette required for the radiosynthesis of [^18^F]FTHA on the Elixys Flex/Chem.

Radiochemical production was performed on the Elixys Flex/Chem (Sofie Biosciences, Dulles, VA, USA), a commercially available automated disposable cassette-based radiosynthesizer. Purification and formulation were performed on the commercially available automated unit, Pure/Form (Sofie Biosciences). The reagent and consumable setup of the cassette is described in Figure 3.

**Figure 4:**
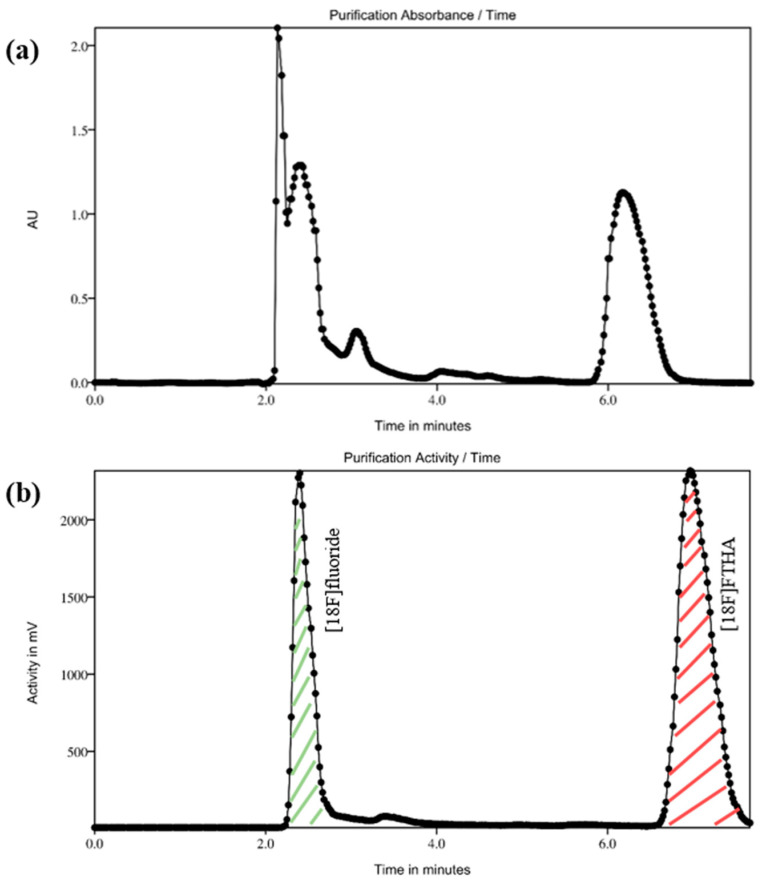
Preparative HPLC chromatograms of [^18^F]FTHA in the cassette-based Elixys synthesizer: (**a**) UV channel (230 nm), (**b**) radioactivity channel.

Synthesis was started with the delivery of [^18^F]fluoride (19–25 GBq) in target water through the PS-HCO_3_- into the cassette using positive pressure (11 psi). Trapped [^18^F]fluoride was subsequently eluted with eluent Solution A (Position 1, Table 5) into the reactor. Iterative azeotropic drying was carried out with stirring under both vacuum and a stream of argon (15 psi) at 110 °C, first for 5 min and the following two times for 4 min. The reactor was cooled to 35 °C, and the precursor solution (Position 4, Table 5) was added. Contents were reacted at 100 °C for 15 min with stirring. Once the reaction was complete, the solution was cooled to 40 °C, and for hydrolysis reaction, 2M KOH (Position 5, Table 5) was added, followed by 4 min of stirring at 50 °C. Neutralized by Solution B (Position 6, Table 5), the reaction mixture was transferred into the HPLC of the Pure/Form. Injecting content went through the connected column Gemini 10 μm C18 110Å 250 × 10 mm, Phenomenex using a mobile phase with a ratio of 850:150:4:2 (*v*/*v*/*v*/*v*) MeOH/H_2_O/AcOH/L–Ascorbic acid and on flow rate of 8 mL/min and UV detector on 230 nm wavelength. Similarly, the average retention time of [^18^F]FTHA was 6–8 min (Figure 4). The product peak was collected into the bulb containing Solution C (Position Bulb, Table 5). The resulting product solution was pushed over the C-18 cartridge into the SPE Waste. The purified product was eluted with EtOH (Position Elute, Table 5) and reconstituted with sterile NaCl 0.9%. (Position Reconstitute, Table 5) in a prefilled product vial with 4 mL of 8% of BSA to achieve a final formulation of 10% EtOH in sterile final product vial (TechneVial 11 mL, Curium, France).

### 4.3. Quality Control

**Figure 5:**
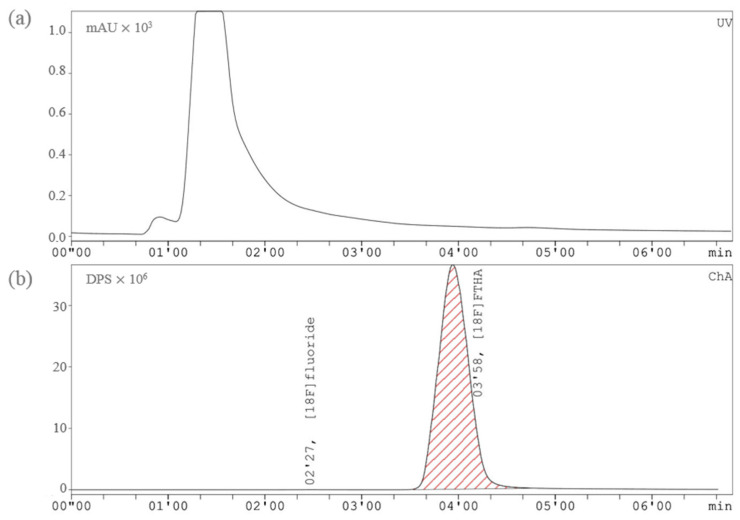
Analytical HPLC chromatograms from formulated [^18^F]FTHA tracer: (**a**) UV channel (230 nm), (**b**) radioactivity channel.

Radioactivity was measured by using a calibrated ionization chamber (VDC-405, Veenstra Instruments, Joure, the Netherlands). Chemical and radiochemical purity (RCP) of [^18^F]FTHA was determined by an analytical HPLC method using the VWR Hitachi (VWR International, Leuven, Belgium), assembled with the Chromaster 5160 pump, the 5410 UV detector (λ = 230 nm), and the Raytest Gabi radiodetector (Raytest, Straubenhardt, Germany). The connected column was the Gemini 10 μm C18 110Å 250 × 4.6 mm (Phenomenex, Torrance, CA, USA), and the mobile phase was 90:10:0.4 (*v*/*v*/*v*) MeOH/H_2_O/AcOH at a flow rate of 1.8 mL/min. Average retention time of [^18^F]FTHA was 4 min (Figure 5). The chemical identity of [^18^F]FTHA was determined by co-injection of the unlabeled reference compound, FTHA (Table 1). All results were integrated with the software GinaStar Elysia-Raytest v 5.9 (Elysia, Straubenhardt, Germany). For gamma spectrometry, a Berthold LB 2045 (Berthold Technologies, Bad Wildbad, Germany) was used. Residual Kryptofix was assessed by a TLC spot test (Celltech K222-TAA) and the solution S from Celltech (Merck, Rahway, NJ, USA). Residual solvents (MeCN and MeOH) were analyzed via gas chromatography Intuvo 900 GC System (Agilent Technologies, Santa Clara, CA, USA); physiochemical parameters (pH and osmolality) were determined with pH stripes pH-Fix 2.0–9.0 (Macherey-Nagel, Düren, Germany) and an osmometer WESCOR VAPRO 5600 (MT Promedt, Ingbert, Germany).

## 5. Conclusions

In summary, we have described our experience with and implementation of the automated radiochemical synthesis of [^18^F]FTHA on two available radiosynthesizer units: a vessel-based synthesizer and the cassette-based Elixys Flex/Chem with a Pure/Form system. We performed a successful transfer to the Elixys module with more than a two-fold increase in the % RCY at EOS using the identical reagent solutions and with compliance with radiochemical, chemical, and physicochemical quality control parameters. The described processes will ensure the reliable availability of [^18^F]FTHA to facilitate clinical and preclinical imaging research studies in the field of lipid metabolism.

## Figures and Tables

**Table 1:** Radiochemical yield of the [^18^F]FTHA syntheses.

Module	*n*	RCY at EOS, GBq (Min–Max)	RCY at EOS, % (Min–Max)	Precursor, mg (Min–Max)	Approx. Duration of Synthesis, mins
Vessel-based synthesizer	46	2.09 ± 0.99 (0.23–4.56)	5.52 ± 2.38 (0.51–10.27)	3.80 ± 0.78 (2.4–5.2)	73
Elixys	12	3.13 ± 1.41 (1.60–6.27)	13.01 ± 5.63 (6.40–25.08)	3.76 ± 0.62 (3.0–4.8)	80

**Table 2:** Quality control results.

Parameters	Method	Acceptance Criteria	Vessel-Based Synthesizer	Elixys
RCP, %	Analytical HPLC	<95	99.26 ± 1.01	99.18 ± 0.77
Radiochemical Identity	Analytical HPLC	Matches retention time of the standard	Yes	Yes
Radionuclidic Purity	Gamma spectrometer	Presence of peak at 511 keV	Yes	Yes
Kryptofix, µg/mL	Kryptofix test	≤50	≤5	≤5
MeCN, ppm	Gas chromatographer	<410	50 ± 40	39 ± 80
MeOH, ppm	Gas chromatographer	<3000	102 ± 50	273 ± 504
pH	pH indicator strip	4.0–8.5	7.1 ± 0.2	6.9 ± 0.2
Osmolality, mosm/kg	Osmometer	200–400	298 ± 45	271 ± 22

**Table 3:** List of used chemicals.

Chemical	Product Number	Company
14-*(R,S)*-[^18^F] Fluoro-6-thia-heptadecanoic acid (Reference standard for [^18^F]FTHA)	2860	ABX (Radeberg, Germany)
Acetic acid (AcOH)	27225	Sigma-Aldrich (Burlington, MA, USA)
Acetonitrile (MeCN)	34851	Sigma-Aldrich (Burlington, MA, USA)
Benzyl-14-*(R,S)*-tosyloxy-6-thiaheptadecanoate (Precursor for [^18^F]FTHA)	2850	ABX (Radeberg, Germany)
Bovine Serum Albumin (BSA)	A7030	Sigma-Aldrich (Burlington, MA, USA)
di-Sodium hydrogen phosphate dihydrate (Na_2_HPO_4_ * 2 H_2_O)	106580	Merck (Rahway, NJ, USA)
Ethanol (EtOH)	100986	Merck (Rahway, NJ, USA)
Kryptofix 222	810647	Merck (Rahway, NJ, USA)
L-Ascorbic acid	A5960	Sigma-Aldrich (Burlington, MA, USA)
Methanol (MeOH)	34860	Sigma-Aldrich (Burlington, MA, USA)
Potassium carbonate (K_2_CO_3_)	791776	Sigma-Aldrich (Burlington, MA, USA)
Potassium hydroxide (KOH)	105032	Merck (Rahway, NJ, USA)
Sodium chloride 9 mg/mL (NaCl 0.9%)	350 5731	B. Braun (Melsungen, Germany)
Sodium dihydrogen phosphate monohydrate (NaH_2_PO_4_ * H_2_O)	106346	Merck (Rahway, NJ, USA)

**Table 4:** List of reagents in the vessel-based synthesizer.

Name of Vial	Amount	Content
Elution vial	0.5 mL	Solution A ^1^
V1	~3.8 mg in 1 mL	Precursor for [^18^F]FTHA in MeCN
V2	0.3 mL	2M KOH
V3	0.63 mL	Solution B ^2^
V4	4.2 mL	NaCl 0.9%
V5	0.8 mL	EtOH
V6	20 mL	Solution C ^3^
Bulb	60 mL	Solution C ^3^

^1^ Solution A: 20 mg Kryprofix 222 and 4.5 mg K_2_CO_3_ in 1 mL 80:20 (*v*/*v*) MeCN:TraceSELECT water. ^2^ Solution B: 30 μL AcOH in 600 μL of the preparative HPLC mobile phase. ^3^ Solution C: 60 mL phosphate buffer (5.1 mg Na_2_HPO_4_ * 2 H_2_O and 2.9 mg NaH_2_PO_4_ * H_2_O in 500 mL B.Braun water (Ecotainer); 0.1 M, pH = 7) + 120 μL ascorbic acid solution (500 mg L-Ascorbic acid in 5 mL B.Braun water (Ecotainer)).

**Table 5:** List of reagents in the Elixys Flex/Chem and Pure/Form.

Position	Amount	Content
Flex/Chem		
1	0.6 mL	Solution A ^1^
2	1 mL	MeCN
3	1 mL	MeCN
4	~3.76 mg in 1 mL	Precursor for [^18^F]FTHA in MeCN
5	0.35 mL	2M KOH
6	0.63 mL	Solution B ^2^
Pure/Form		
Bulb	60 mL	Solution C ^3^
Rinse	6 mL	Solution C ^3^
Elute	0.8 mL	EtOH
Reconstitute	3.2 mL	NaCl 0.9%

^1^ Solution A: 20 mg Kryprofix 222 and 4.5 mg K_2_CO_3_ in 1 mL 80:20 (*v*/*v*) MeCN:TraceSELECT water. ^2^ Solution B: 30 μL AcOH in 600 μL of the preparative HPLC mobile phase. ^3^ Solution C: 60 mL phosphate buffer (5.1 mg Na_2_HPO_4_ * 2 H_2_O and 2.9 mg NaH_2_PO_4_ * H_2_O in 500 mL B.Braun water (Ecotainer); 0.1 M, pH = 7) + 120 μL ascorbic acid solution (500 mg L-Ascorbic acid in 5 mL B.Braun water (Ecotainer)).

## Data Availability

Data is contained within the article.
